# Self-reported adherence of ambulance nurses to acute chest pain guidelines in Southern Sweden: a cross-sectional study

**DOI:** 10.1136/bmjopen-2025-110199

**Published:** 2026-01-22

**Authors:** Michael Ulrich Hansen, Malin Axelsson, Vedrana Vejzovic, Ulf Jakobsson, Slobodan Zdravkovic

**Affiliations:** 1 Department of Care Science, Faculty of Health and Society, Malmö universitet, Malmö, Sweden; 2 Clinical Science, Clinical Research Centre, Malmo, Sweden

**Keywords:** Cross-Sectional Studies, Guideline Adherence, CARDIOLOGY, Clinical Decision-Making, Clinical Protocols, Nurses

## Abstract

**Abstract:**

**Objective:**

To examine the self-reported adherence of ambulance nurses to acute chest pain guidelines and analyse how demographic and professional characteristics influence this adherence.

**Design:**

Cross-sectional study.

**Setting:**

Regional ambulance service in southern Sweden (18 ambulance stations).

**Participants:**

Ambulance nurses (registered and specialist nurses). Of the 397 ambulance nurses invited, 261 responded (65.7%) in 2023.

**Data analysis:**

Descriptive statistics; independent-samples t-tests and χ^2^ tests for group comparisons; Pearson correlation; and stepwise linear regression to identify predictors of adherence.

**Primary and secondary outcome measures:**

Primary: adherence to the prehospital acute chest pain guideline, measured with the 5-item Self-Reported Adherence scale (5–25). Secondary: medication-specific adherence; guideline-access sources.

**Method:**

A cross-sectional study involving 261 ambulance nurses from 18 ambulance stations in southern Sweden. Adherence to acute chest pain guidelines was assessed using a validated instrument. Data collected in autumn 2023 were analysed using descriptive and inferential statistics, including stepwise linear regression analysis.

**Results:**

The study revealed an average self-reported adherence score of 19.2 out of 25 for acute chest pain guidelines. Mobile applications were the most commonly used source for accessing acute chest pain guidelines, while ambulance managers were the least used. Notably, older and more experienced ambulance nurses reported higher adherence scores. Additionally, a positive attitude towards the guidelines was correlated with higher adherence. Prioritisation of guidelines and age were predictors of adherence. In contrast, other demographic variables, such as sex and specialist nursing education, were not found to be associated with adherence.

**Conclusion:**

The study indicates that self-reported adherence to acute chest pain guidelines among ambulance nurses is influenced by how highly they prioritise these guidelines and by their attitudes towards them, as well as their age and professional experience. Enhancing educational programmes and digital resources, particularly for younger and less experienced nurses, may improve adherence and patient outcomes in prehospital settings.

**STRENGTHS AND LIMITATIONS OF THIS STUDY**

- Large, region-wide sample of ambulance nurses across 18 emergency medical services (EMS) stations with a 65.7% response rate, enhancing internal precision.
- Used an EMS-specific, psychometrically evaluated self-report measure of adherence to acute chest pain guidelines.
- Adherence was self-reported, which may overestimate actual behaviour.
- Cross-sectional design limits causal inference between identified factors and adherence.
- The single-region Swedish setting and local practice patterns may limit generalisability to other EMS systems.

## Introduction

 Acute chest pain is a common symptom within prehospital emergency services, accounting for 10%–21% of emergency medical services (EMS) encounters.^1^,^4^ Of these, 10%–15% involve life-threatening conditions, especially myocardial infarction (MI),^5^,^7^ thus necessitating prompt and precise diagnosis to enhance patient outcomes. Ambulance nurses, often the first point of professional medical contact, are pivotal in the early recognition and management of MI, typically relying on clinical presentation and 12-lead ECG findings.^8^ Evidence-based protocols recommended by the European Society of Cardiology (ESC)^8^ emphasise the early prehospital administration of acetylsalicylic acid (aspirin) to reduce mortality and enhance efficacy.^8 9^

Despite the recognised benefits of guidelines,^10^ adherence among ambulance nurses is inconsistent for acute chest pain. When MI is confirmed, ambulance nurses are more likely to adhere to the guidelines for pharmaceutical treatment.^5^ However, overall adherence to guidelines, including the administration of aspirin, is substantially lower compared with other EMS guidelines,^11^ with observed rates ranging from 7.8% to 58% of all MI encounters.^59 12^,^14^ This issue raises significant concerns due to its association with elevated mortality rates.^9^

In general, issues such as staffing shortages and training deficiencies,^15 16^ teamwork challenges and colleague disagreement,^15 17^ insufficient outcome-related feedback^18 19^ and the complexity of the guidelines^17 18 20^ further hinder adherence. Adherence is also affected by individual decision-making and professional judgement, which depend on clinical experience and familiarity with protocols.^4 18 20^ Professional confidence, often undermined by gaps in medical training that fail to emphasise essential nursing and contextual skills, further complicates adherence.^21^ As nurses gain experience, their reasoning may shift from analytical to intuitive, thus sometimes deviating from guidelines.^18 20 22^ Additional factors such as lack of awareness, disagreement with guidelines^14 17 23 24^ and variations in professional roles—including differences between general nurses and specialist nurses—further undermine effective practice.^23^ These issues highlight demographic and professional factors that impede effective practice.^19^ Moreover, although ambulance nurses use resources such as formal education, digital tools, workplace training and feedback to navigate clinical challenges, the specific usage and impact of these resources on adherence to acute chest pain guidelines remain unclear.^17 20^

However, most research on guideline adherence in prehospital care is based on retrospective analyses of EMS medical records.^5 9 12 13^ Such data can quantify whether recommended treatments were documented but provide limited insights into the reasoning ability of ambulance nurses and the contextual factors that shape their decisions. In addition, previous research aggregates adherence across different EMS guidelines, which makes it difficult to disentangle challenges specific to acute chest pain, where adherence rates appear particularly low.^59 12^,^15^ To our knowledge, no previous study has specifically examined the self-reported adherence of ambulance nurses to acute chest pain guidelines. The present study addresses this gap by examining the self-reported adherence of ambulance nurses and exploring how demographic and professional characteristics are associated with this self-reported adherence.

## Objective

This study aimed to examine the self-reported adherence of ambulance nurses to acute chest pain guidelines and analyse how demographic and professional characteristics influence this adherence.

## Methods

### Study design and setting

This cross-sectional study was conducted in the Skåne region, southern Sweden, a region covering both rural and urban areas with a population of approximately 1.4 million. The EMS system responds to calls requiring ambulance care, coordinated through a dispatch centre. Ambulance nurses provide acute care, including examination (vital signs, pain score and 12-lead ECG), treatment (aspirin, nitroglycerin, morphine and oxygen) and transport, delivered according to the regional guideline aligned with ESC recommendations.^8^ This study adhered to the ‘Strengthening the Reporting of Observational Studies in Epidemiology’ guidelines for cross-sectional studies.^25^

### Participants

Participants were required to be registered nurses with a 3-year Bachelor of Science in Nursing. Some participants had also completed a specialist nursing education, which entails an additional 1–2 years of master’s-level education in a specific field, but this was not a requirement for study participation. At the start of the questionnaire, participants were asked to confirm that they were familiar with the regional guideline for acute chest pain; only those who indicated familiarity were eligible to complete the questionnaire.

For this study, general and specialist nurses are collectively referred to as ambulance nurses. In the study region, ambulances are staffed by at least one ambulance nurse (a registered nurse, often with specialist education) who has on-scene clinical responsibility and administers treatment in accordance with regional guidelines. Sampling occurred consecutively during work shifts and educational events from 6 September 2023 to 18 December 2023, at 18 ambulance stations across the region. Coordination with the ambulance managers ensured that all potential participants were invited. In total, 397 eligible ambulance nurses were invited, and the study was designed as a census of the target population.

### Data collection

In this study, adherence was measured using the Self-Reported Adherence instrument, specifically tailored to measure self-reported adherence to regional guidelines for managing acute chest pain, that are aligned with ESC guidelines.^8^ The instrument comprises five items rated on a five-point scale (never–always; total score 5–25). One general item asks: “How often do you follow the guideline when caring for patients with acute chest pain in ambulance care?” The remaining four items ask how often participants administer aspirin, nitroglycerin, morphine and oxygen according to the acute chest pain guidelines when caring for patients with acute chest pain. In the instrument introduction, the Self-Reported Adherence items were described as referring to the regional acute chest pain guidelines used for patients with acute chest pain in situations where the guideline is clinically applicable (ie, patients with undifferentiated chest pain in whom suspected acute coronary syndrome is being considered). Higher scores indicate higher self-reported adherence to this guideline. The Self-Reported Adherence instrument has demonstrated acceptable reliability and validity among ambulance nurses through psychometric analysis.^26^

Demographic and professional background information was collected through self-report, including age, sex, years of experience as a nurse, years of experience as a specialist nurse, years of experience in prehospital care and specialist nursing education. Data were also collected on sources through which participants accessed guidelines information (education, mobile application, the Internet, colleagues, ambulance managers, ambulance physicians and research). Participants were additionally asked to rate how highly they prioritised the acute chest pain guideline in their daily practice (from ‘very low’ to ‘very high’) and their attitude towards this acute chest pain guideline (from ‘very negative’ to ‘very positive’).

The Self-Reported Adherence instrument and background items were administered as a paper-based questionnaire. Questionnaires were distributed during work shifts and educational sessions at all 18 ambulance stations in the region (including satellite posts). The participants could complete the questionnaire immediately on site or later at their convenience and return it in a sealed envelope. Completing the instrument typically took around 15 min. The completed questionnaires were assigned unique random identifiers and digitised by an independent external firm not affiliated with the research team before being transferred to the researchers for analysis.

### Data analysis

Descriptive statistics were used to present demographic characteristics, including frequencies, means, SD and percentages. Group differences were analysed with independent-samples t-tests (eg, sex, specialist nursing education and years of experience categories). Assumptions were assessed prior to analysis: normality via Shapiro–Wilk tests and Q–Q plots, and homogeneity of variances via Levene’s test.

Further analyses explored the associations between adherence and various sources of guideline information, as well as how the prioritisation of and attitude towards these guidelines among ambulance nurses influenced their adherence scores. The association between age and adherence scores was examined using Pearson’s correlation coefficient. Additionally, Pearson’s χ^2^ test was used to analyse the proportion of men and women with specialist nursing education.

Stepwise linear regression analysis was used to identify significant predictors of adherence, incorporating variables based on their statistical contribution. Data analysis was conducted using SPSS V.28.0,^27^ and reported with two-sided p values together with 95% CIs; statistical significance was set at p<0.05. Missing values were excluded to ensure data completeness.

### Ethical considerations

The Swedish Review Authority approved the study (Dnr: 2019-02112/2021-03368), and all methods were conducted in accordance with the Declaration of Helsinki involving human participants.^28^ Before participation, participants were provided with detailed oral and written information about the study. In line with ethical approval, informed consent for the cross-sectional survey was obtained from participants by completion and return of the anonymous questionnaire, which was regarded as provision of written consent. Each completed instrument was assigned a unique, randomly generated identifier and processed by a third party firm not connected to the research group. All analyses were subsequently performed by the research team related to the study, who maintained confidentiality in handling and accessing the data.

## Results

### Demographic characteristics

The study invited 397 ambulance nurses, with 261 completing the questionnaire, resulting in a 65.7% response rate. The sample comprised 120 men (46.0%) and 141 women (54.0%). The majority (77.4%) were specialist nurses, with 29.9% having more than 10 years of experience as specialist nurses and 35.6% having over 10 years of experience in prehospital care (table 1). The average age of participants was 40.9 years (±9.3), with men averaging 42.6 years (±9.4) and women 39.5 years (±9.0), a statistically significant difference (p=0.007). Specialist nurses were older (average 42.5 years) compared with general nurses (average 35.3 years, p<0.001). However, there were no statistically significant differences in education between men and women (χ^2^=0.399, p=0.528).

**Table 1:** Demographic characteristics by gender

Variables		Total n=261 (%)	Men n=120 (%)	Women n=141 (%)
Education	General nurses	59 (22.6)	25 (20.8)	34 (24.1)
	Specialist nursing education	202 (77.4)	95 (79.2)	107 (75.9)
Nursing specialist education	Prehospital emergency care	148 (56.7)	71 (59.2)	77 (54.6)
	Anaesthetic care	34 (13)	24 (20)	10 (7.1)
	Intensive care	23 (8.8)	7 (5.8)	16 (11.3)
	Other nursing specialist education*	17 (6.5)	4 (3.3)	13 (9.2)
Multiple nursing specialist education	One nursing specialist education	182 (90.1)	84 (70)	98 (69.5)
	Two nursing specialist education	20 (9.9)	11 (9.2)	9 (6.4)
Employment status	Part-time employment	7 (2.7)	6 (5)	1 (0.7)
	Full-time employment	252 (97.3)	112 (94.9)	140 (99.3)
Professional experience general nursing	<1 year	1 (0.4)	0 (0)	1 (0.7)
	1–3 years	16 (6.2)	6 (5)	10 (7.1)
	4–6 years	48 (18.5)	15 (12.6)	33 (23.4)
	7–9 years	48 (18.5)	23 (19.3)	25 (17.7)
	≥10 years	147 (56.5)	75 (63)	72 (51.1)
Professional experience specialist nursing	<1 year	18 (8.9)	9 (9.5)	9 (8.4)
	1–3 years	42 (20.8)	17 (17.9)	25 (23.4)
	4–6 years	31 (15.3)	9 (9.5)	22 (20.6)
	7–9 years	33 (16.3)	17 (17.9)	16 (15)
	≥10 years	78 (38.6)	43 (45.3)	35 (32.7)
Professional experience prehospital emergency care	<1 year	26 (10)	10 (8.3)	16 (11.3)
	1–3 years	61 (23.4)	23 (19.2)	38 (27)
	4–6 years	45 (17.2)	18 (15)	27 (19.1)
	7–9 years	36 (13.8)	17 (14.2)	19 (13.5)
	≥10 years	93 (35.6)	52 (43.3)	41 (29.1)

* Other nursing specialist education: emergency care nurses, midwives, psychiatric nurses, surgical nurses and theatre nurses

### Adherence among ambulance nurses

**Figure 1:**
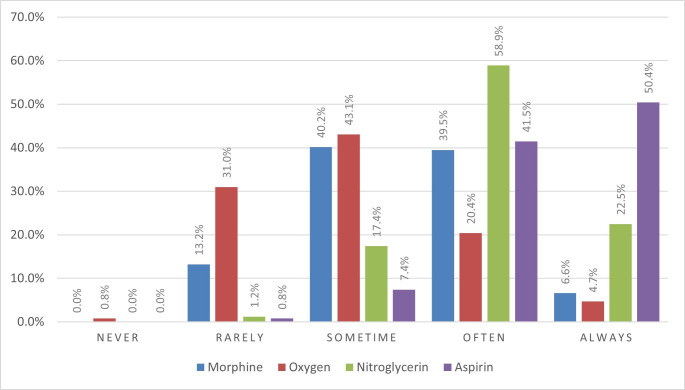
Self-reported proportions of medications administered by ambulance nurses to patients with acute chest pain.

The self-reported adherence of ambulance nurses to administering treatment for acute chest pain varied across four medications. Aspirin and nitroglycerin were the most administered, with 50.4% and 22.5% of respondents, respectively, reporting that they always use them. In contrast, oxygen and morphine were used less consistently, with only 4.7% and 6.6% of participants, respectively, indicating that they always administer these drugs (figure 1).

### Adherence to guidelines by demographic and professional characteristics

The overall mean adherence score was 19.2 out of a possible 25 points, with scores ranging from 14 to 25. Analysis of adherence scores by sex indicated no significant difference, with males scoring an average of 19.37 (±2.44) and females scoring 19.10 (±2.03), resulting in a p value of 0.328. A positive correlation was found between age and adherence scores (r=0.178, p=0.004), indicating that adherence increased with age. There were no significant differences in adherence scores between general nurses and those with specialist nursing education (p=0.322; table 2). Additionally, there were no statistically significant differences in adherence scores among nurses with different specialist nursing education: ambulance care (p=0.213), anaesthesia (p=0.671), intensive care (p=0.057) or other specialist nursing education (p=0.571). Moreover, holding multiple specialist nursing educations compared with specialist education also did not significantly impact adherence score (p=0.090).

**Table 2:** Self-reported adherence to acute chest pain guidelines by demographic and professional characteristics

Variables	Adherence score, mean (SD)	Mean difference (95% CI)	P value*
Sex			0.335
Male	19.37 (2.44)		
Female	19.10 (2.03)	0.27 (−0.28 to 0.83)	
Education			0.322
General nurses	19.48 (2.24)		
Specialist nurses	19.15 (2.22)	−0.33 (−0.99 to 0.33)	
Employment status			0.897
Full-time employment	19.21 (2.24)		
Part-time employment	19.33 (1.51)	0.12 (−1.70 to 1.94)	
Years of experience as general nurse			**0.035**
<10 years	18.89 (2.37)		
≥10 years	19.48 (2.09)	−0.59 (−1.15 to −0.04)	
Years of experience as specialist nurse			**0.019**
<10 years	18.86 (2.23)		
≥10 years	19.62 (2.14)	−0.76 (−1.39 to −0.12)	
Years of experience in prehospital emergency care			0.119
<10 years	19.07 (2.30)		
≥10 years	19.52 (2.06)	−0.46 (−1.03 to 0.12)	
Prioritisation towards guidelines			**<0.001**
Very low – moderate	16.18 (1.55)		
High – very high	19.45 (2.11)	−3.28 (−4.30 to −2.25)	
Attitude towards guidelines			**0.015**
Very negative – neutral†	17.87 (2.23)		
Quite positive – very positive‡	19.31 (2.21)	−1.45 (−2.60 to −0.29)	

* Independent samples t-test; mean difference and 95% CI between categories. P values are reported in bold; values <0.05 were considered statistically significant

† Dichotomised variable: attitude towards the acute chest pain guideline; ‘very negative–neutral’ versus ‘quite positive–very positive’

‡ Dichotomised variable: prioritisation of the acute chest pain guideline; ‘very low–medium’ versus ‘high–very high’

Regarding professional experience, nurses with more than 10 years as general or specialist nurses reported significantly higher adherence scores than those with less than 10 years, with p values of 0.035 and 0.019, respectively (table 2). In contrast, no significant differences in adherence scores were observed for prehospital care experience between those with less than 10 years of experience and those with 10 or more years of experience (p=0.119). Furthermore, adherence scores were significantly higher among those who prioritised guidelines (p<0.001) and held a positive attitude toward guidelines (p=0.015).

### The impact of diverse information sources on guideline adherence

**Figure 2:**
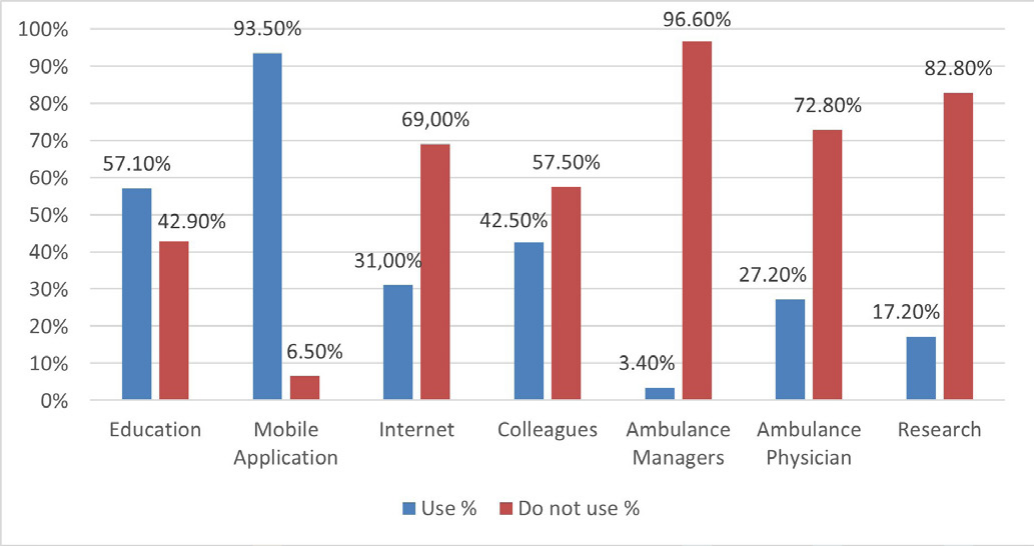
Information sources for guidelines used by ambulance nurses.

Among the 261 participants, 93.5% primarily used mobile applications to access the guidelines, supplemented by education (9.6%) and interaction with colleagues (5.4%; figure 2). Only 1.9% of respondents reported not using any sources for guidelines. Most participants accessed 1–7 sources, typically combining 3 sources (28.0%). No significant differences were found in adherence scores based on the type of source used.

### The influence of demographic characteristics on adherence score

A stepwise linear regression model showed that high prioritisation of the acute chest pain guidelines was the strongest predictor, increasing adherence scores by 3.463 points (B=3.463, p<0.001; table 3). Age was also a significant predictor, with each additional year increasing adherence scores by 0.061 points (B=0.061, p<0.001). The model demonstrated an adjusted coefficient of determination R^2^ of 0.228 for Model 2, indicating that the predictors accounted for approximately 22.8% of the variance in adherence scores. The other investigated variables were not identified as predictors of self-reported adherence.

**Table 3:** Stepwise linear regression analysis of demographic and professional characteristics influencing adherence score

Included variables	B	Coefficient standard error	95% CI for B	P value
Model 1				
Constant (adherence score)	13.324	1.342	10.68 to 16.00	**<0.001**
Age	0.056	0.024	0.01 to 0.10	**0.018**
Sex (men, women)	−0.439	0.296	−1.02 to 0.15	0.140
Employment status (full-time, part-time)	0.168	0.832	−1.47 to 1.81	0.840
Nursing experience (<10, ≥10 years)	0.261	0.412	−0.55 to 1.07	0.528
Specialist nursing experience (<10, ≥10 years)	0.239	0.450	−0.65 to 1.13	0.595
Prehospital emergency care experience (<10, ≥10 years)	−0.457	0.467	−1.38 to 0.47	0.330
Attitude towards guidelines*	0.157	0.568	−0.96 to 1.28	0.783
Prioritisation towards guidelines†	3.595	0.556	2.50 to 4.70	**<0.001**
Model 2				
Constant (adherence score)	13.396	0.845	11.73 to 15.06	**<0.001**
Prioritisation towards guidelines†	3.463	0.510	2.46 to 4.47	**<0.001**
Age	0.061	0.016	0.03 to 0.09	**<0.001**

Model 1 includes all presented variables; Model 2 is a result of the stepwise linear regression; P values in bold indicate statistical significance (p<0.05)

* Dichotomised variable: attitude towards the acute chest pain guideline (‘very negative–neutral’ versus ‘quite positive–very positive’)

† Dichotomized variable: prioritization of the acute chest pain guideline (‘very low–medium’ versus ‘high–very high’)

## Discussion

This study aimed to examine the self-reported adherence of ambulance nurses to acute chest pain guidelines and analyse how demographic and professional characteristics influence this adherence. Results highlight that prioritisation, positive attitudes, age and experience were associated with higher adherence. Older and more experienced ambulance nurses also had higher scores, underlining experience as a key factor in guideline application. Furthermore, mobile applications were the most reported source for accessing guidelines, while ambulance managers were the least used.

Previous pre-hospital studies have reported guideline adherence estimates between 7.8% and 58%.^59 12^,^14^ In comparison, the self-reported adherence scores in our study are towards the upper end of this range, although differing measures and definitions of adherence limit direct comparisons. Importantly, our study does not observe guidelines adherence directly; instead, it captures the perceptions of ambulance nurses of how often they follow the acute chest pain guidelines. Self-reported measures are practical but may overestimate adherence because of recall error and social desirability bias.^29 30^ In contrast, observational or record-based methods primarily capture document behaviours and may still miss the underlying reasoning or appropriate deviations from the guideline. Psychometric scales such as the Self-Reported Adherence measure provide insight into perceived behaviours and underlying attitudes,^31^ but they do not offer a direct measure of actual adherence in clinical practice. For this reason, our findings should be interpreted as describing self-reported adherence rather than objective adherence, and future research is needed to link self-reported adherence to patient-level clinical data. It remains important to discuss how adherence to the medication recommendations was operationalised. The four items on aspirin, nitroglycerin, morphine and oxygen ask how often these drugs are used for patients with suspected acute heart disease in ambulance care, but they do not include clinical indications or reasons for withholding a drug, such as allergy or active bleeding (aspirin), hypotension or suspected right ventricular infarction (nitroglycerin), reduced level of consciousness or respiratory depression (morphine), or normal oxygen saturation (oxygen).^8^ Consequently, lower reported use may sometimes represent appropriate care rather than non-adherence, and our findings should therefore be interpreted as self-reported adherence rather than a precise measure of correct or incorrect medication use at the patient level.

The findings further revealed that older nurses and those with longer experience reported higher adherence scores, suggesting that professional experience tends to align with better adherence to established guidelines.^32 33^ However, it is crucial to recognise that while work experience can enhance adherence, it can also lead to deviations from guidelines, as experienced nurses may prioritise their judgement over strict protocols.^18 20^ Despite extensive prehospital care experience, this did not correspond to higher adherence scores, indicating that prolonged work experience may not always translate to better adherence.^15^ This paradox underscores the complex relationship between experience and adherence. Interestingly, our study revealed that specialist nursing education alone does not significantly impact adherence scores. This finding aligns with research showing that postgraduate education alone is insufficient to guarantee enhanced guideline adherence,^17 34 35^ underscoring the need for complementary strategies.

Our results show that nurses with a positive attitude towards the acute chest pain guidelines and who report prioritising its application consistently achieve higher self-reported adherence scores. This finding is consistent with other studies, showing that favourable perceptions of guidelines are linked to better adherence and patient outcomes.^15 23 36^ Emphasising the practical benefits and critical importance of following guidelines can significantly enhance adherence.^37^ Notably, pre-hospital studies have shown that favourable attitudes and supportive implementation are associated with higher adherence to protocols.^15 38^ The impact of the attitudes and knowledge of nurses appears to influence adherence; those with negative views or insufficient knowledge often struggle with guideline implementation.^39 40^ This variation highlights the significant role of organisational culture and sociocultural beliefs in relation to adherence,^17^ which aligns with identified barriers to guideline adherence in EMS.^15^ While our study highlights the importance of attitude and prioritisation, further research is needed to investigate the impact of organisational culture and sociocultural beliefs on guideline adherence.

Mobile applications were the primary source of guidelines information for 93.5% of ambulance nurses, which may be explained by their accessibility during clinical practice. In contrast, ambulance managers, ambulance physicians and research were less frequently used as sources. There was no single source; nurses drew on multiple channels, and differences between channels may influence perceived usability, attitudes and adherence. Although digital tools have been proven to enhance decision support and promote guideline adherence among ambulance nurses,^41^ they also present challenges that require active involvement to integrate quality and safety measures into clinical practice effectively.^42^ Relying solely on mobile applications may not be adequate for increasing adherence to guidelines. Combining these with education interventions, along with audit and feedback,^43^ may significantly enhance their effectiveness, as our results indicate that education is a frequently used information source. Notably, younger physicians have shown promising results in accessing information about guidelines, medical education and training using mobile applications.^44 45^ This strategy could be beneficially applied to younger and less experienced ambulance nurses to enhance their adherence to guidelines.

Future research should therefore build on our findings by combining the Self-Reported Adherence instrument with event-based data collection at individual patient encounters and qualitative interviews. Such mixed-methods designs would make it possible to document when and why the guideline is not followed, to distinguish justified flexibility from problematic non-adherence, and to compare self-reported adherence with observed practice and patient outcomes.

## Strengths and limitations

While our study benefits from a robust sample size and a comprehensive validated instrument,^26^ which instils confidence in the reliability of our findings, several limitations must be acknowledged. First, the use of self-reported data may introduce recall and social desirability bias, and our results therefore reflect perceived rather than observed adherence.^46^ Additionally, our study did not apply categorical thresholds (low/moderate/high) because none have been validated for this instrument.^26^ Second, the cross-sectional design restricts the ability to infer causal relationships between the observed factors and self-reported adherence. Third, the study was conducted within a Swedish, nurse-based EMS system in which ambulances are predominantly staffed by registered nurses, often with specialist education. Many other EMS systems internationally are paramedic-based with different educational requirements and scope of practice, which may limit the transferability of our findings. Moreover, we did not have access to individual level data on non-responders and therefore could not perform a formal non-response analysis. Although the response rate of 65.7% is relatively high, we cannot rule out selection bias: nurses who are more engaged with or positive towards guidelines may have been more likely to participate, whereas those with lower adherence may be under-represented. This potential limitation should be considered when interpreting and applying our findings.

## Conclusion

Self-reported adherence to acute chest pain guidelines among ambulance nurses appears to be influenced by how highly they prioritise these guidelines, by having more positive attitudes toward them, as well as by age and professional experience. Given the widespread use of mobile applications for accessing guidelines, enhancing these digital tools through targeted educational interventions and continuous feedback is crucial for improving adherence-supportive behaviours. Effectively integrating such technology may foster a supportive culture that values adherence to guidelines, particularly by strengthening positive attitudes among younger and less experienced nurses. Although our findings are based on self-reported adherence and a single regional EMS system, they highlight potentially modifiable targets for interventions that aim to improve guideline use and, ultimately, patient outcomes in prehospital settings.

## Data Availability

Data are available upon reasonable request.
