# Supplementary material for: Self-reported adherence of ambulance nurses to acute chest pain guidelines in Southern Sweden: a cross-sectional study
Source: BMJ Open. 2026 Jan 22;16(1):e110199. doi: 10.1136/bmjopen-2025-110199 (PMC12829355; doi:10.1136/bmjopen-2025-110199)
Supplement: Supplementary file 1 [file bmjopen-2025-110199.reviewer_comments.pdf]

PEER REVIEW HISTORY

BMJ Open publishes all reviews undertaken for accepted manuscripts. Reviewers are asked to complete a checklist review form and are provided with free text boxes to elaborate on their assessment. These free text comments are reproduced below.

ARTICLE DETAILS

Title (Provisional)

Ambulance Nurses' Self-Reported Adherence to Acute Chest Pain Guidelines in Southern Sweden: A Cross-Sectional Study

Authors

Hansen, Michael Ulrich; Axelsson, Malin; Vejzovic, Vedrana; Jakobsson, Ulf; Zdravkovic, Slobodan

---

VERSION 1 - REVIEW

**Reviewer**	**1**
**Name**	**Wibring, Kristoffer**
**Affiliation**	**University of Gothenburg Institute of Health and Care Sciences**
**Date**	**16-Sep-2025**
**COI**	**None**

Thank you for the opportunity to review this manuscript on ambulance nurses' self-perceived guideline adherence in the care of patients with chest pain. This is a well-written manuscript that provides novel insights into self-reported guideline adherence, complementing previous research, which has predominantly relied on retrospective analyses of EMS medical records. It would be of considerable interest to also include such objective data and analyses, as this would allow a comparison between self-reported adherence and adherence as documented in the EMS medical records. I understand, however, that access to such data may not have been available.

Title

I suggest that the title is changed into: Ambulance Nurses' Self-Assessed Adherence to Acute Chest Pain Guidelines: A Cross-Sectional Study

Abstract

-

Strengths and limitations – bullet points

-

Introduction

P3 L70 – It may be more appropriate to describe acute chest pain as a symptom rather than a condition.

P4 L101-104 – Consider rephrasing these sentences, as their current wording does not convey the intended meaning with sufficient clarity. Please, rephrase these sentences for improved readability.

Objective

The term ‘assess’ may not be the most appropriate choice. You may wish to consider whether, for example, examine’ would more accurately reflect the study’s methodology and findings.

Methods

P4 L119-121 – You may wish to consider rephrasing this sentence to clarify that specialist nursing education was not a requirement. For example: “Participants were required to be registered nurses with a three-year Bachelor of Science in Nursing, some of whom had completed an additional one to two years of master’s-level education in a specific field. However, this was not a requirement for study participation.”

P4 L121 – How was this familiarity confirmed? In writing when filling in the Self-Reported Adherence instrument?

P4 L128-130 – Please report the minimum detectable effect size implied by your sample size calculation.

P5 L131-146 – Please provide more detailed information on how data were collected using the Self-Reported Adherence instrument. Was it completed digitally or on paper? How long did it typically take to complete? Were participants supervised while completing the instrument, or could they fill it in later at their convenience? Providing these details is important to enable replication of the study in other settings.

P5 L 159. “Finally,” can be removed.

Results

Table 2, please provide confidence intervals instead of p-value.

Table 2, the a could be placed in the p-value heading (or confidence interval heading, if updated) instead of being repeated on every line.

Table 3, please provide confidence intervals instead of p-value.

Discussion

P10 L233-238 – It may be more accurate to state that adherence is not assessed, but rather that self-reported adherence is rated or described. You may wish to consider rephrasing this

or elaborating on what is meant by the assessment. It is also important to underline that your data and findings do not provide information on actual guideline adherence, but only on the self-reported experience of adherence. The current phrasing may imply that it reflects actual adherence.

Conclusion

P11 L287 – I would suggest that you change into “Self-perceived adherence to acute chest pain guidelines…”

Strengths and limitations

I am not fully acquainted with the BMJ Open author guidelines; however, in most scientific journals the Strengths and Limitations section is typically presented before the Conclusion.

P11 L301-302 – Not least, EMS personnel have different educational backgrounds. For example, in Sweden and the Netherlands, EMS staff are primarily nurses, whereas in many other countries, EMS systems are based on paramedics with varying levels of education.

What about potential bias or skewness in terms of which nurses choose to participate versus those who decline? Is it possible that these two groups differ systematically? There may be a risk that nurses with higher guideline adherence are more willing to participate, whereas those with lower adherence are less likely to do so. This potential selection bias should be acknowledged and discussed.

**Reviewer**	**2**
**Name**	**Ferry, Amy**
**Affiliation**	**University of Edinburgh, Cardiovascular Science**
**Date**	**23-Sep-2025**
**COI**	**None**

Comments to the author

This paper focuses on an important question as care for conditions such as acute chest pain is transferred out of the hospital setting to a pre-hospital setting in an attempt to ensure early delivery of appropriate care. This of course can only be achieved if clinical guidelines are adhered to. I have questions about the methods used to answer the research question outlined below.

Suggestions for improvement

Major

I require further information about the context in which the Self-Reported Adherence questions were asked. “How often do you follow the guideline when caring for patients with acute chest pain in ambulance care?” If the EMS nurse has assessed the patient and

concludes there is an obvious alternative diagnosis to the chest pain (something other than suspected ACS) then not following the ESC guideline may be the appropriate action. Were the questions asked in the context of suspected acute coronary syndrome or undifferentiated chest pain?

Similarly, I require more information about how the medication frequency items were asked. Administration of oxygen is not associated with clinical benefit if oxygen levels are >90% therefore it is not recommended. Was the question 'how often do you administer oxygen?', or 'how often do you administer oxygen if SaO2 is >90% /<90%'? These questions would be answered differently.

Again for morphine – I would be concerned if every patient had morphine administered when they were not in severe pain so not administering morphine is the appropriate action and should not be termed non-adherence.

I suggest the Self-Reported Adherence scale is included as an appendix so the reader is better informed.

I feel this question could have been better answered by asking EMS nurses to complete the scale at actual patient attendances with options to state why the guideline was not followed (which could be entirely appropriate). I accept the authors state that observational studies may only capture explicit behaviours but employing a mixed methods approach (e.g. qualitative interviews with a subset of participants) would have allowed the authors to capture deeper insights into guideline adherence and compare to actual self-reported data.

Prioritisation and attitude to guidelines were identified as predictors of adherence. Was this to guidelines in general or specifically the chest pain guideline?

VERSION 1 - AUTHOR RESPONSE

**Reviewer: 1**<br><br>**Mr. Kristoffer Wibring, University of Gothenburg Institute of Health and Care Sciences**	
Thank you for the opportunity to review this manuscript on ambulance nurses' self-perceived guideline adherence in the care of patients with chest pain. This is a well-written manuscript that provides novel insights into self-reported guideline adherence, complementing previous research, which has predominantly relied on retrospective analyses of EMS medical records. It would be of considerable interest to also include such objective data and analyses, as this would allow a comparison between self-reported adherence and adherence as documented in the EMS medical records. I understand, however, that access to such data may not have been available.	We thank the reviewer for taking the time to read our manuscript carefully and for the thorough and constructive review, as well as the positive comments about our work. We appreciate the thoughtful suggestions for improvement. Below, we address each comment point by point.<br><br>We appreciate the reviewer's suggestion to compare self-reported adherence with EMS medical records, as this would indeed provide valuable insights. Unfortunately, such data were not available for the present study, as noted in the review. Nevertheless, we recognize the importance of this comparison and will take it into consideration in the design of future research.
Title	We agree that it is important to emphasize the self-

I suggest that the title is changed into: Ambulance Nurses' Self-Assessed Adherence to Acute Chest Pain Guidelines: A Cross-Sectional Study	reported nature of the outcome in the title. Adherence to the acute chest pain guidelines was measured using the Self-Reported Adherence instrument. To emphasize the self-reported nature of the outcome and, in line with the Editor's suggestion, we have also added location in the title: Ambulance Nurses' Self-Reported Adherence to Acute Chest Pain Guidelines in Southern Sweden: A Cross-Sectional Study (P1, 1-3).
Introduction<br>P3 L70 – It may be more appropriate to describe acute chest pain as a symptom rather than a condition.	We agree and have rephrased the opening sentence of the introduction to describe acute chest pain as a symptom instead of a condition:<br><br>Acute chest pain is a common symptom within prehospital emergency services, accounting for 10-21% of emergency medical services (EMS) encounters [1-4]. (P3, 69-70).
Introduction<br><br>P4 L101-104 – Consider rephrasing these sentences, as their current wording does not convey the intended meaning with sufficient clarity. Please, rephrase these sentences for improved readability.	We appreciate this comment and have rewritten the paragraph describing previous guidelines-adherence research to improve clarity and explicitly distinguish between retrospective record-based studies and our focus on self-reported adherence:<br><br>However, most research on guideline adherence in prehospital care is based on retrospective analyses of EMS medical records [5,9,12,13]. Such data can quantify whether recommended treatments were documented but provide limited insights into ambulance nurses' reasoning and the contextual factors that shape their decisions. In addition, previous research aggregate adherence across different EMS guidelines, which makes it difficult to disentangle challenges specific to acute chest pain, where adherence rates appear particularly low [5,9,12–15]. To our knowledge, no previous study has specifically examined ambulance nurses' self-reported adherence to acute chest pain guidelines. The present study addresses this gap by examining ambulance nurses' self-reported adherence and exploring how demographic and professional characteristics are associated with this self-reported adherence. (P3-4, 96-104).
Objective<br>The term 'assess' may not be the most appropriate choice. You may wish to consider whether, for example, examine' would more accurately reflect the study's methodology and findings.	We agree that "examine" better reflects our design. We have changed "assess" to "examine" in the abstract and objective:<br><br>This study aimed to examine ambulance nurses' self-reported adherence to acute chest pain guidelines and analyze how demographic and professional characteristics influence this adherence. (P2, 29-30; P4, 106-107).
Methods<br><br>P4 L119-121 – You may wish to consider rephrasing this sentence to clarify that specialist nursing education was not a requirement. For example: "Participants were	We agree with the reviewer and have rephrased the description of participant eligibility to clarify that specialist nursing education was not required. The text now reads:

required to be registered nurses with a three-year Bachelor of Science in Nursing, some of whom had completed an additional one to two years of master's-level education in a specific field. However, this was not a requirement for study participation."	Participants were required to be registered nurses with a three-year Bachelor of Science in Nursing. Some participants had also completed a specialist nursing education, which entails an additional one to two years of master's – level education in a specific field, but this was not a requirement for study participation. (P4, 118-121)
Methods<br><br>P4 L121 – How was this familiarity confirmed? In writing when filling in the Self-Reported Adherence instrument?	We have clarified this in the participants' subsection:<br><br>At the start of the questionnaire, participants were asked to confirm that they were familiar with the regional guideline for acute chest pain; only those who indicated familiarity were eligible to complete the questionnaire. (P4, 121-123)
Methods<br><br>P4 L128-130 – Please report the minimum detectable effect size implied by your sample size calculation.	We thank the reviewer for this helpful comment. In the original submission, we reported a sample size calculation. On reflection, we have collected data through a census sample and received a response rate of 65.7%. Under such a census design, a power analysis is not considered applicable. To avoid confusion, we have removed the sample size calculation from the Methods section and now clarify the design as follows:<br><br>In total, 397 eligible ambulance nurses were invited, and the study was designed as a census of the target population. (P4, 129-130).
Methods<br><br>P5 L131-146 – Please provide more detailed information on how data were collected using the Self-Reported Adherence instrument. Was it completed digitally or on paper? How long did it typically take to complete? Were participants supervised while completing the instrument, or could they fill it in later at their convenience? Providing these details is important to enable replication of the study in other settings.	We have expanded the description of data collection procedures for the Self-Reported Adherence instrument:<br><br>The Self-Reported Adherence instrument and background items were administered as a paper-based questionnaire. Questionnaires were distributed during work shifts and educational sessions at all 18 ambulance stations in the region (including satellite posts). The participants could complete the questionnaire immediately on site or later at their convenience and return it in a sealed envelope. Completing the instrument typically took around 15 minutes. (P5, 151-155)
Methods<br><br>P5 L 159. "Finally," can be removed.	We have removed the word "Finally" from the last sentence in the Data analysis subsection:<br><br>Data analysis was conducted using SPSS version 28.0,… (P6, 170)
Results<br>Table 2, please provide confidence intervals instead of p-value.	We agree that including confidence intervals improves interpretability. We have revised Table 2 so that it now represents mean adherence scores (with standard deviations) for each group together with 95% confidence intervals for the mean difference between groups.<br><br>Please see Table 2 (P9, 224-226).

Results<br><br>Table 2, the a could be placed in the p-value heading (or confidence interval heading, if updated) instead of being repeated on every line.	We agree and have now updated the table so that the superscript 'a' appears in the p-value heading instead of being repeated for each value. Please see Table 2 (P9, 224-226).
Results<br><br>Table 3, please provide confidence intervals instead of p-value.	We have revised Table 3 to report the regression coefficients (B) together with their 95% confidence intervals, in line with BMJ Open emphasis on estimates and precision. P values are retained as secondary information. Please see Table 3 (P10, 242-248)
Discussion<br>P10 L233-238 – It may be more accurate to state that adherence is not assessed, but rather that self-reported adherence is rated or described. You may wish to consider rephrasing this or elaborating on what is meant by the assessment. It is also important to underline that your data and findings do not provide information on actual guideline adherence, but only on the self-reported experience of adherence. The current phrasing may imply that it reflects actual adherence.	We agree with the reviewer and have revised the Discussion to clarify that the study describes self-reported, rather than observed, adherence. The paragraph now explicitly states that "our study does not observe guideline adherence directly; instead, it captures ambulance nurses' self-report of how often they follow the acute chest pain guidelines" and that "our findings should be interpreted as describing self-reported adherence rather than objective adherence." See changes:<br><br>This study aimed to examine ambulance nurses' self-reported adherence to acute chest pain guidelines and analyze how demographic and professional characteristics influence this adherence. (P11, 250-251).<br><br>Previous prehospital studies have reported guideline adherence estimates between 7.8% and 58% [5,9,12–14]. In comparison, the self-reported adherence scores in our study are toward the upper end of this range, although differing measures and definitions of adherence limit direct comparisons. Importantly, our study does not observe guidelines adherence directly; instead, it captures ambulance nurses' perceptions of how often they follow the acute chest pain guidelines. Self-reported measures are practical but may overestimate adherence because of recall error and social desirability bias [29,30]. In contrast, observational or record-based methods primarily capture document behaviors and may still miss the underlying reasoning or appropriate deviations from the guideline. Psychometric scales such as the Self-Reported Adherence measure provide insight into perceived behaviors and underlying attitudes [31], but they do not offer a direct measure of actual adherence in clinical practice. (P11, 256-265).
Conclusion<br>P11 L287 – I would suggest that you change into "Self-perceived adherence to acute chest pain guidelines…"	We thank the Reviewer for this helpful suggestion. Adherence to the acute chest pain guidelines was measured using the Self-Reported Adherence instrument, which is a self-report instrument. To reflect this operationalization of the outcome and to be consistent with the title and Methods section, we have chosen to use the term "self-reported adherence." The first sentence of the conclusion now reads:<br><br>Self-reported adherence to acute chest pain guidelines among ambulance nurses appears to be influenced by

	how highly they prioritize these guidelines, by having more positive attitudes toward them, as well as by age and professional experience. (P13, 333-335)
Strengths and limitations<br><br>I am not fully acquainted with the BMJ Open author guidelines; however, in most scientific journals the Strengths and Limitations section is typically presented before the Conclusion.	We have moved the narrative Strength and limitations section so that it now precedes the Conclusion section, in line with your suggestion and common journal structure (P12-13, 316-331).
P11 L301-302 – Not least, EMS personnel have different educational backgrounds. For example, in Sweden and the Netherlands, EMS staff are primarily nurses, whereas in many other countries, EMS systems are based on paramedics with varying levels of education.	We have expanded the Strengths and limitations section to explicitly acknowledge that the Swedish EMS system is mainly nurse-based (registered nurses, often with specialist education), while many other national EMS systems are paramedic-based with differing levels and types of education. Please see:<br><br>Third, the study was conducted within a Swedish, nurse-based EMS system in which ambulances are predominantly staffed by registered nurses, often with specialist education. Many other EMS systems internationally are paramedic-based with different educational requirements and scope of practice, which may limit the transferability of our findings. (P12, 323-326).
What about potential bias or skewness in terms of which nurses choose to participate versus those who decline? Is it possible that these two groups differ systematically? There may be a risk that nurses with higher guideline adherence are more willing to participate, whereas those with lower adherence are less likely to do so. This potential selection bias should be acknowledged and discussed.	Thank you for highlighting this important issue. We agree that there is a risk of selection bias if nurses who chose to participate differ systematically from those who declined. Unfortunately, we did not have access to individual level data on non-responders and were therefore unable to perform a formal non-response analysis to compare respondents and non-respondents. We now clarify this limitation in the Strengths and limitations section. The revised text reads:<br><br>Moreover, we did not have access to individual level data on non-responders and therefore could not perform a formal non-response analysis. Although the response rate of 65.7% is relatively high, we cannot rule out selection bias: nurses who are more engaged with or positive towards guidelines may have been more likely to participate, whereas those with lower adherence may be underrepresented. This potential limitation should be considered when interpreting and applying our findings. (P12-13, 327-332)
**Reviewer: 2**<br><br>**Dr. Amy Ferry, University of Edinburgh**	**Authors' comments**
This paper focuses on an important question as care for conditions such as acute chest pain is transferred out of the hospital setting to a pre-hospital setting in an attempt to ensure early delivery of appropriate care. This of course can only be achieved if clinical guidelines are adhered to. I have questions about the methods used to answer the research question outlined below.	Thank you very much for your careful review and for highlighting important conceptual and methodological concerns regarding how guideline adherence is measured. We are grateful that you took the time to read our manuscript in detail and for your thorough and constructive comments, as well as the positive feedback about our work. We appreciate your thoughtful suggestions for improvement. Below, we address each comment point by point.

I require further information about the context in which the Self-Reported Adherence questions were asked. "How often do you follow the guideline when caring for patients with acute chest pain in ambulance care?" If the EMS nurse has assessed the patient and concludes there is an obvious alternative diagnosis to the chest pain (something other than suspected ACS) then not following the ESC guideline may be the appropriate action. Were the questions asked in the context of suspected acute coronary syndrome or undifferentiated chest pain?	We agree that the clinical context of the Self-Reported Adherence items should be clarified. In the Methods, Data collection section, we have added a sentence specifying that the items refer to the regional acute chest pain guideline:<br><br>In this study, adherence was measured using the Self-Reported Adherence instrument, specifically tailored to measure self-reported adherence to regional guidelines for managing acute chest pain, that are aligned with ESC guidelines [8]. The instrument comprises five items rated on a five-point scale (never-always; total score 5-25). One general item asks: "How often do you follow the guideline when caring for patients with acute chest pain in ambulance care?" The remaining four items ask how often participants administer aspirin, nitroglycerine, morphine and oxygen according to the acute chest pain guidelines when caring for patients with acute chest pain. In the instrument introduction, the Self-Reported Adherence items were described as referring to the regional acute chest pain guidelines used for patients with acute chest pain in situations where the guideline is clinically applicable (i.e. patients with undifferentiated chest pain in whom suspected acute coronary syndrome is being considered). Higher scores indicate higher self-reported adherence to this guideline. The Self-Reported Adherence instrument has demonstrated acceptable reliability and validity among ambulance nurses through psychometric analysis [26]. (P5, 132-143)
Similarly, I require more information about how the medication frequency items were asked. Administration of oxygen is not associated with clinical benefit if oxygen levels are >90% therefore it is not recommended. Was the question 'how often do you administer oxygen?', or 'how often do you administer oxygen if SaO_2 is >90% /<90%'? These questions would be answered differently.<br><br>Again for morphine – I would be concerned if every patient had morphine administered when they were not in severe pain so not administering morphine is the appropriate action and should not be termed non-adherence.	We thank the reviewer for this important clarification request. In the instrument introduction, respondents were informed that these items referred to the regional acute chest pain guideline used when caring for patients with acute chest pain and suspected acute coronary syndrome. Thus, they were asked to indicate how often they perceived that they administered each medicine in accordance with this guideline; the items themselves do not specify particular thresholds (e.g. SaO_2) or other clinical indications. Please see revised text for clarification:<br><br>The remaining four items ask how often participants administer aspirin, nitroglycerine, morphine and oxygen according to the acute chest pain guidelines when caring for patients with acute chest pain. In the instrument introduction, the Self-Reported Adherence items were described as referring to the regional acute chest pain guidelines used for patients with acute chest pain in situations where the guideline is clinically applicable (i.e. patients with undifferentiated chest pain in whom suspected acute coronary syndrome is being considered). (P5, 136–141).<br><br>We also note in the Discussion section that these items do not capture oxygen saturation, pain intensity or contraindications; therefore, lower reported use of

	oxygen or morphine may sometimes represent appropriate care rather than non-adherence:<br><br>It remains important to discuss how adherence to the medication recommendations was operationalized. The four items on aspirin, nitroglycerine, morphine and oxygen ask how often these drugs are used for patients with suspected acute heart disease in ambulance care, but they do not include clinical indications or reasons for withholding a drug, such as allergy or active bleeding (aspirin), hypotension or suspected right ventricular infarction (nitroglycerine), reduced level of consciousness or respiratory depression (morphine), or normal oxygen saturation (oxygen)[8]. Consequently, lower reported use may sometimes represent appropriate care rather than non-adherence, and our findings should therefore be interpreted as self-reported adherence rather than a precise measure of correct or incorrect medication use at the patient-level. (P11, 267-275)
I suggest the Self-Reported Adherence scale is included as an appendix so the reader is better informed.	We thank the reviewer for this helpful suggestion. The Self-Reported Adherence instrument, which was used to assess adherence, is currently available only in Swedish, and an English version has not yet been translated and psychometrically tested. For this reason, we have not included the full questionnaire as an appendix. Instead, we have clarified and expanded the description of the instrument in the Methods, Data collection section (P5, 132-143), including its structure, number of items, response alternatives, and scoring, so that readers are better informed.
I feel this question could have been better answered by asking EMS nurses to complete the scale at actual patient attendances with options to state why the guideline was not followed (which could be entirely appropriate). I accept the authors state that observational studies may only capture explicit behaviours but employing a mixed methods approach (e.g. qualitative interviews with a subset of participants) would have allowed the authors to capture deeper insights into guideline adherence and compare to actual self-reported data.	We agree that asking EMS nurses to complete the scale at actual patient attendances and combining this with qualitative interviews would provide richer insights into guideline adherence and reasons for deviating from the guideline. We have previously conducted a qualitative study that explored how ambulance nurses use the acute chest pain guideline in clinical practice; the results from this study are summarised in the Background section and the full paper is cited in the reference list (Ulrich Hansen et al. 2022 [18]). However, the present study was deliberately designed as a cross-sectional survey to examine self-reported adherence and associations with individual factors in a large sample; event-level data collection and interviews were beyond the scope and resources of this project. We have now made this limitation explicit in the Discussion:<br><br>Future research should therefore build on our findings by combining the Self-Reported Adherence instrument with event-based data collection at individual patient encounters and qualitative interviews. Such mixed-methods designs would make it possible to document when and why the guideline is not followed, to distinguish justified flexibility from problematic non-adherence, and to compare self-reported adherence with observed practice and patient outcomes. (P12, 311-315).

Prioritisation and attitude to guidelines were identified as predictors of adherence. Was this to guidelines in general or specifically the chest pain guideline?	Thank you for this question. Both variables refer specifically to the regional acute chest pain guideline, not to clinical guidelines in general. In the questionnaire, participants were asked: (1) how highly they prioritised the acute chest pain guideline in their daily practice (from "very low" to "very high"), and (2) their overall attitude towards this acute chest pain guideline (from "very negative" to "very positive"). We have clarified this wording in the Data collection section (P5, 148-150), and the footnotes to Tables 2 (P9, 226) and 3 (P10, 246-248) specify that prioritisation and attitude were dichotomised items referring to the acute chest pain guideline.

VERSION 2 - REVIEW

Reviewer 1

Name Wibring, Kristoffer

Affiliation University of Gothenburg Institute of Health and Care Sciences

Date 18-Dec-2025

COI

I would like to commend the authors for revising the manuscript in a highly satisfactory manner. All my previous comments have been carefully addressed, and the revisions have substantially improved the clarity and methodological transparency of the manuscript. After reviewing the manuscript once more, as well as the authors' responses to the comments, I have no further questions or comments.
